# Irritable Bowel Syndrome

**DOI:** 10.1155/2012/612479

**Published:** 2012-05-07

**Authors:** Per G. Farup, Ami D. Sperber, Magnus Simrén

**Affiliations:** ^1^Unit for Applied Clinical Research, Faculty of Medicine, Norwegian University of Science and Technology, 7491 Trondheim, Norway; ^2^Department of Research, Innlandet Hospital Trust, 2819 Gjøvik, Norway; ^3^Faculty of Health Sciences, Ben-Gurion University of the Negev, Beer-Sheva 84105, Israel; ^4^Department of Internal Medicine, Institute of Medicine, Sahlgrenska Academy, University of Gothenburg, 41345 Gothenburg, Sweden

Studies of Irritable Bowel Syndrome (IBS) and other biopsychosocial disorders show a great variety of approaches to important clinical challenges. The papers in this special issue shed light on new and interesting aspects of IBS. 

IBS has a surprisingly uniform worldwide distribution, from east to west and north to south, and affects males and females and all age groups [1, 2]. The prevalence rates vary from 2% to 22% and the disorder is everywhere related to a high degree of comorbidity (psychiatric distress, muscle-skeletal complaints, etc.), increased use of health care resources, and environmental factors (early life events, familial factors, infections, diet, social factors, etc.) [2, 3]. In this issue, the study by A. López-Colombo et al. “*The epdimology of functional gastrointestinal disorders in Mexico: a population-based study*” confirmed this pattern in the first population-based study on functional gastrointestinal disorders in Mexico. IBS had a prevalence rate of 16% and was associated with depression and increased use of health care resources. 

A substantial proportion of patients with IBS report onset of their symptoms after acute gastroenteritis [4], and in a recent meta-analysis it was demonstrated that the risk of developing IBS following infectious gastroenteritis was increased sixfold [5]. Several risk factors for developing postinfectious IBS have been demonstrated, related to the pathogen or the infection and genetic, clinical or environmental factors [6]. However, the majority of the published studies so far have included subjects living in the industrialized world, and it is therefore not clear if the same relationship between gastrointestinal (GI) infections and the development of long-standing GI symptoms exists in the developing world. In these countries the inhabitants are exposed to acute and chronic GI infections to a larger extent, beginning already in infancy. Therefore, the contribution of Morgan et al. “*Irritable bowel syndrome and gastrointestinal parasite infection in a developing nation environment*” in this issue of Gastroenterology Research and Practice is a welcome contribution to this research area. The authors included a large group of subjects from a population-based sample in Nicaragua, and studied one group of subjects with IBS and a randomly selected group without IBS. Parasite carriage in fecal samples, which may serve as a surrogate for repeated exposures to gastrointestinal pathogens and infection, was investigated to assess the association between repeated GI infections and IBS. The main finding in this well-performed study was that no difference in parasite carriage could be demonstrated between IBS cases (16.6%) and controls (15.4%), implicating that the strong link between GI infections and development of IBS seen in investigations from the industrialized countries may not be applicable to developing countries. However, prospective studies in well-defined cohorts are needed in order to further assess this relationship, and to evaluate if post-infectious IBS is a phenomenon specific to areas where GI infections are not as common as in developing countries. It is tempting to speculate that this may be related to different immunological responses to GI infections based on the background prevalence of infections consistent with hygiene hypothesis, but this remains to be proven [7].

IBS and other functional gastrointestinal disorders are characterized by a high rate of psychosocial comorbidity as well as comorbidity with functional somatic syndromes such as fibromyalgia and chronic pelvic pain [8, 9]. These high rates have been attributed to peripheral and central hypersensitivity [10], which is modulated, in part, by stress and psychological disorders such as anxiety, depression, and somatization [11]. Three papers in this issue relate to these subjects. The paper by M.-J. Gerson and C. Gerson “*The importance of relationships in patients with irritable bowel syndrome*” reviews the effect of a patient's relationships (family, friends, work) on the IBS illness experience. It concludes that although this is a subject that has not received sufficient attention in the medical literature and requires rigorous confirmation, relationships do have an impact on the IBS illness experience and may even have an effect on response to treatment. Doctors should inquire whether family members and intimate others are sufficiently educated about IBS, and whether they are supportive to the patient or whether there are problems that can and should be addressed. A. S. Butt et al. “*Irritable bowel syndrome and psychiatric disorders in Pakistan: a case control study*” conducted a study in Pakistan to estimate the frequency and strength of association of common mental disorders (anxiety and depression) in patients with IBS and controls. This is of particular interest because there is scant information in the medical literature on the significance of this association in Asian countries. The authors found that these disorders are more common in patients with IBS in Pakistan, as in western countries, compared to other chronic diseases including migraine and hypertension. L. B. Olafsdottir et al. “*Natural history of irritable bowelsyndrome in women and dysmenorrhea: a 10-year follow-up study*” from Iceland conducted a 10-year longitudinal study to assess associations between IBS and dysmenorrhea before and after menopause. The authors concluded that women with IBS are more likely to experience dysmenorrhea than women without IBS and that IBS symptom severity seems to increase after menopause. Although these papers focus on different specific associations between IBS and common psychosocial and comorbid conditions, taken together they add to the body of information on these relationships with IBS and do so from countries such as Pakistan and Iceland from which there is relatively little available information.

Treatment of IBS includes a general therapeutic approach, lifestyle advice with dietary recommendations, psychosocial support and pharmacotherapy, which all are effective [3]. However, since most treatments benefit only a minority, the treatment should be tailored on an individual basis. IBS is a long-lasting, often life-long, disorder with an undulating course and high placebo response rate. Despite the chronicity of the disorder, most therapeutic trials are of rather short duration, commonly 3–6 months and show a high rate of rapidly appearing relapses. S. Evangelista *“Benefits from long term treatment in irritable bowel syndrome”* gives in this issue a good overview of the benefits from long-term treatment and takes into account the natural course of the disorder, the placebo effect, and the relapse rates related to type and duration of the treatment. It is likely that prolongation of the treatment should be reserved for patients with recurrent symptoms, and that cyclic treatment might be appropriate for most patients due to the undulating natural course of the disorder.

Despite years of research, today's health care for patients with IBS still merits improvement based on better understanding of the disorder [12]. The papers included in this issue adds important new information to the IBS puzzle, but we probably still have a long way to go and many studies to perform before truly effective treatments will be available.

*Per G. Farup* *Per G. Farup* *Ami D. Sperber* *Ami D. Sperber* *Magnus Simrén* *Magnus Simrén*
